# Association Between Riboflavin Intake and Telomere Length: A Cross-Sectional Study From National Health and Nutrition Examination Survey 1999–2002

**DOI:** 10.3389/fnut.2022.744397

**Published:** 2022-03-31

**Authors:** Weihua Chen, Shanshan Shi, Yizhou Jiang, Liling Chen, Ying Liao, Kaihong Chen, Kun Huang

**Affiliations:** ^1^Longyan First Affiliated Hospital of Fujian Medical University, Longyan, China; ^2^The Third Clinical Medical College, Fujian Medical University, Fuzhou, China; ^3^State Key Laboratory of Cardiovascular Disease, Fuwai Hospital, National Center for Cardiovascular Diseases, Chinese Academy of Medical Sciences, Peking Union Medical College, Beijing, China; ^4^Center for Statistical Science, Department of Industrial Engineering, Tsinghua University, Beijing, China

**Keywords:** riboflavin, dietary intake, telomere length, NHANES, obese, female

## Abstract

**Background:**

Dietary habits and dietary intake affect telomere length, a reliable marker of biological aging and a predictor of chronic disease. Riboflavin (RF) is known as a water-soluble antioxidant vitamin, but its role in telomere length maintenance has yet to be elucidated.

**Objective:**

The purpose of this study was to examine the relationship between dietary RF intake and telomere length in a nationally representative sample of adults.

**Methods:**

Using the NHANES (1999–2002), telomere data of 4,298 participants aged ≥45 years were analyzed in a cross-sectional manner. Leukocyte telomere length was measured using the quantitative real-time polymerase chain reaction (qPCR). Dietary RF intake was assessed by a trained interviewer using 24-h dietary recall method. Generalized linear regressions were performed to evaluate the association between dietary RF intake and telomere length. Subgroup analyses were performed to further explore this relationship in sex and body mass index (BMI) subgroups.

**Results:**

Among the 3,788 participants included, the average telomere length was longer in females (*P* = 0.014), while they had a lower average RF intake compared to males (*P* < 0.001). There was a weak positive correlation between RF intake and telomere length both when unadjusted (β = 0.011; *P* = 0.037) and adjusted for age, sex, and ethnicity (β = 0.013; *P* = 0.033). Subgroup analyses showed a positive association between RF intake and the telomere length in female after adjusting for confounding factors (β = 0.029; *P* = 0.046). In the female subgroup, there were significant positive relationships between telomere length and RF intake in the obese group (β = 0.086, *P* = 0.022).

**Conclusion:**

Increased dietary RF intake was significantly associated with longer telomere length in middle-aged and older American females, especially in low RF intake obese female.

## Introduction

Riboflavin (RF) was first discovered by Cardoso DR in 1879 as a yellow pigment found in milk (1). RF is also a water-soluble and heat-stable essential vitamin, as well as an often-neglected anti-oxidant (2). Studies have shown that higher dietary RF intake showed beneficial anti-aging (3) and anti-cancer effects (4, 5), while insufficient RF intake was associated with increased risks of various diseases, including anemia (6) and cardiovascular diseases (7).

Studies have related telomere length with biological aging (8). The shortening of telomere might imply higher risks of cardiovascular diseases (9), cancer (10), and other metabolic diseases (11). Both endogenous (for example, genetics, inflammation, and DNA damage) and environmental (for example, smoking, alcohol, and life stress) factors could be involved in telomere maintenance and regulation (12). Evidence from human studies suggested that oxidative stress may accelerate telomere shortening in human cells, whereas antioxidants have the potential to retard this process (13, 14). Given the fact that the amount of food and nutrient intake of antioxidants (including vitamin C and vitamin E) may help maintain telomere length (15, 16), RF intake as part of a normal diet may also support the fight against oxidative stress (2). Therefore, it is natural to hypothesize that increased dietary RF intake is associated with telomere lengthening. However, studies on the relationship between dietary RF intake and telomere length were rare.

In this study, we used data from a large population-based survey, the National Health and Nutrition Examination Survey (NHANES), to investigate the relationship between dietary RF intake and telomere length in middle-aged and older adults.

## Materials and Methods

### Study Populations

The NHANES is a nationally representative cross-sectional survey in the United States, recursively conducted by the National Center for Health Statistics (NCHS) every 2 years, and for 5,000 sampled individuals each survey. The survey obtained written informed consent from all participants, and approval from Ethics Review Board of the NCHS (Protocol #98e12) (available on the web at: NHANES -- National Health and Nutrition Examination Survey Homepage^1^).

**FIGURE 1:**
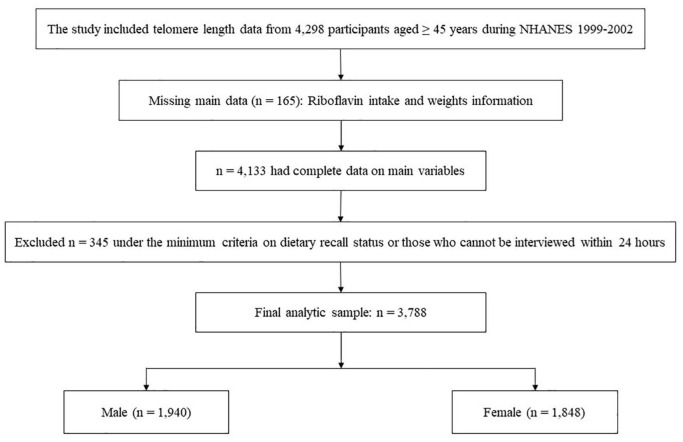
NHANES 1999–2002 analytic sample flow diagram.

The study included telomere length data from 4,298 participants aged ≥45 years during the 1999–2000 and 2001–2002 NHANES cycles. We excluded participants under the minimum criteria on dietary recall status, with missing weights information or those who cannot interview within 24 h. The recall record was verified as reliable and met the following minimum criteria for the overall quality and completeness of the reported dietary information: (1) Less than 25% foods with missing descriptive information (e.g., caffeinated or decaffeinated, preparation methods, or brand names); (2) Less than 15% foods with missing amounts; and Any meal reported must have at least one known food. For example, if a respondent reported having a lunch but could not remember any foods from that lunch, the recall did not meet the criterion [available on the web at: NHANES – National Health and Nutrition Examination Survey Homepage (see text footnote 1)]. In the end, a total of 3,788 participants were enrolled in the present study (Figure 1).

### Telomere Length Measurement

National Health and Nutrition Examination Survey collected blood samples from all surveyed individuals. Real-time quantitative polymerase chain reaction (qPCR) was used to measure telomere length relative to standard reference DNA (T/S ratio) in the blood leukocytes. Each sample was assigned to duplicate wells in 96-well plate and was assayed three times on 3 different days. To normalize between-run variability, each assay plate contained 8 control DNA. The inter-assay coefficient of variation was 6.5%. Full details about the telomere length measurement are available on the web at: https://wwwn.cdc.gov/Nchs/Nhanes/2001-2002/TELO_B.htm.

### Riboflavin Intake Assessment

National Health and Nutrition Examination Survey obtained dietary intake information (including types and amounts of foods and beverages consumed in a 24-h period) through What We Eat in America (WWEIA), a series of 24-h dietary recall interviews conducted in the Mobile Examination Center (MEC). The intakes of dietary RF and other food components were estimated based on the University of Texas Food Intake Analysis System and U.S. Department of Agriculture (USDA) Survey Nutrients Database. The nutrient estimates did not include nutrients obtained from dietary supplements or medications. Detailed descriptions of the dietary data collection were provided in the NHANES Dietary Interviewers Procedure Manuals^2^.

### Statistical Analysis

We used the NHANES recommended weights to account for planned oversampling of certain groups. The continuous variables were expressed as means ± standard deviations, and the categorical variables were presented as counts (percentages). Baseline characteristics between the two sex groups were compared using the *t*-test for continuous variables and the χ^2^ test for categorical variables. The relationship between dietary RF intake and telomere length was assessed using a linear regression model (LRM). Three LRMs were applied in the present study, with adjustment for potential confounders ascertained based on prior publications (17–21). Model 1 was a crude model unadjusted for potential confounders. Model 2 was adjusted for demographic factors, including age, sex, and ethnicity. To avoid the adverse impact of multicollinearity on the regression model, we collected information from literature on the correlation between anti-oxidants and other explanatory variables, and filters out variables that has a high correlation with another variable (correlation ≥ 0.50, *p*-value < 0.050) (Supplementary Table 1). Finally, Model 3 was further adjusted for smoking status, alcohol consumption, education style, body mass index (BMI), coronary heart disease (CHD), congestive heart failure (CHF), diabetes mellitus (DM), vitamin C intake, vitamin B12 intake, selenium intake, zinc intake, copper intake, and dietary fiber intake. In addition, we carried out restricted cubic spline analyses to explore the non-linear dose-response relationship between dietary RF intake and telomere length in the whole participants, and three knots were placed at the 5th, 50th, and 95th percentiles. Since previous studies have shown that obesity and sex are associated with telomere length (22, 23), subgroup analyses were performed to assess the potential modifying effects of participant sex and BMI-categorized obesity levels, including normal (BMI < 25 kg/m^2^), overweight (25–29.9 kg/m^2^), obese (BMI in 30.0–34.9 kg/m^2^), and severe-obese (BMI ≥ 35.0 kg/m^2^). Moreover, we explored whether there was an effect of obesity level (non-obese: BMI < 30 kg/m^2^; obese group: BMI ≥ 30 kg/m^2^) on the relationship between RF intake and telomere length in the sex subgroup by adding an interaction term. Finally, we divided RF into a high RF intake group and a low RF intake group based on the first weighted quartile (1.18 mg) and investigated the relationship between obesity (non-obese: BMI < 30 kg/m^2^; obese group: BMI ≥ 30 kg/m^2^) and different RF intake levels in the female subgroup. A two-sided *p*-value < 0.05 indicated significance for all analyses. All data analyses were performed using Survey package in R software (version 4.0.4; R Foundation for Statistical Computing, Vienna, Austria).

## Results

### Clinical Characteristics

Table 1 provided a description of the basic characteristics of the study participants by sex. There were 3,788 participants in the final analysis sample. The average age of the participants was 60.38 ± 0.33 years. The average telomere length is longer in female than in male (0.96 ± 0.02 vs. 0.99 ± 0.02, *P* = 0.014). On the contrary, compared to male, the female had a lower RF and vitamin C intake (2.32 ± 0.04 vs. 1.79 ± 0.04, *P* < 0.001; 99.57 ± 3.46 vs. 91.74 ± 3.17, *P* = 0.006).

**TABLE 1:** Sample size and weighted characteristics of NHANES 1999–2002.

	Total (*n* = 3,788)	Male (*n* = 1,940)	Female (*n* = 1,848)	*P* value
Characters	Weighted distributions of the participants			
Age, years	60.38 ± 0.33	59.54 ± 0.30	61.15 ± 0.43	<0.001
Ethnicity, n (%)				
Mexican American	692 (3.5)	354 (3.7)	338 (3.3)	0.093
Other Hispanic	186 (5.2)	92 (5.5)	94 (4.9)	
White (non-Hispanic)	2206 (80.3)	1139 (80.4)	1067 (80.2)	
Non-Hispanic black	620 (8.1)	317 (8.1)	303 (8.2)	
Other	84 (2.9)	38 (2.3)	46 (3.4)	
Education levels				
Less than high school	1356 (22.6)	698 (21.3)	658 (23.9)	0.004
Completed high school	863 (25.6)	404 (23.5)	459 (27.5)	
More than high school	1565 (51.8)	837 (55.2)	728 (48.6)	
Alcohol consumption, n (%)				
<12/year	2142 (99.0)	1288 (98.5)	1000 (99.5)	0.135
≥12/year	30 (1.0)	31 (1.5)	5 (0.5)	
BMI, kg/m^2^	28.53 ± 0.21	28.33 ± 0.17	28.71 ± 0.28	0.096
<25	1024 (29.7)	495 (26.0)	529 (33.1)	<0.001
25–29.9	1408 (37.7)	822 (44.2)	586 (31.7)	
30–34.9	735 (19.4)	373 (20.1)	362 (18.7)	
≥35	473 (13.3)	173 (9.8)	300 (16.4)	
Smoking, n (%)	2051 (54.1)	1291 (65.0)	760 (44.0)	<0.001
CHD, n (%)	278 (7.03)	188 (8.92)	90 (5.30)	0.005
CHF, n (%)	188 (4.26)	98 (4.20)	90 (4.31)	0.898
DM, n (%)	535 (11.14)	281 (11.51)	254 (10.80)	0.485
Riboflavin intake, mg/d	2.05 ± 0.03	2.32 ± 0.04	1.79 ± 0.04	<0.001
Vitamin C intake, mg/d	95.49 ± 3.05	99.57 ± 3.46	91.74 ± 3.17	0.006
Vitamin B12 intake, mg/d	5.06 ± 0.18	5.91 ± 0.24	4.27 ± 0.26	<0.001
Selenium intake, mcg/d	101.98 ± 1.32	119.85 ± 1.92	85.56 ± 1.39	<0.001
Zinc intake, mg/d	11.27 ± 0.19	13.12 ± 0.24	9.57 ± 0.22	<0.001
Copper intake, mg/d	1.27 ± 0.02	1.44 ± 0.03	1.12 ± 0.02	<0.001
Dietary fiber intake, gm/d	16.10 ± 0.36	17.84 ± 0.49	14.49 ± 0.31	<0.001
Telomere length, T/S ratio	0.97 ± 0.02	0.96 ± 0.02	0.99 ± 0.02	0.014

*NHANES, National Health and Nutrition Examination Survey; BMI, body mass index; CHD, Coronary heart disease; CHF, Congestive heart failure; DM, Diabetes mellitus. Mean and standard deviation were presented for continuous variables, number and proportion were presented for categorical variables.*

At baseline, there was no significant difference in the proportion of males and females of all ethnicities (*P* = 0.093). In contrast, the number of males with higher levels of education was significantly higher than that of females (*P* = 0.004). The percentage of non-obese people in this population was 67.4%, while the number of people with obese was similar in two groups (20.1 vs. 18.7%), and the percentage of severe-obese people was higher in female than in male (16.4 vs. 9.8%). Smoking rates were high among males (65.0 vs. 44.0%, *P* < 0.001).

### Primary Outcomes

**FIGURE 2:**
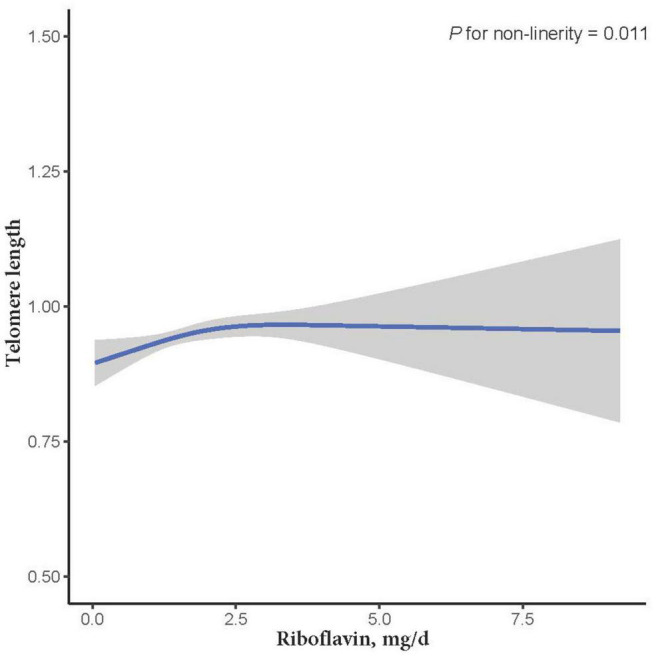
The dose-response relationship between dietary riboflavin intake and telomere length. Point estimates (solid line) and 95% confidence intervals (dashed lines) were estimated by restricted cubic splines analysis with knots placed at the 5th, 50th, and 95th percentile (minimum as the reference).

The relationship between dietary RF intake and telomere length is presented in Table 2. In the crude model, RF intake was positively associated with telomere length (β = 0.011; 95% CI: 0.001, 0.021; *P* = 0.037). After adjusting for age, sex, and ethnicity in the second LRM model, the association remained significant with a slightly increased magnitude (β = 0.012; 95% CI: 0.002, 0.023; *P* = 0.033). However, after adjusting for other control factors (education levels, smoking, alcohol consumption, vitamin C intake, vitamin B12 intake, selenium intake, zinc intake, copper intake, dietary fiber intake, BMI, CHD, CHF, and DM), the association between RF intake and telomere length became insignificant (β = 0.014; 95% CI: −0.002, 0.029; *P* = 0.107; Table 2). In addition, we further used the restricted cubic splines to estimate the dose-response relationship between dietary RF intake and telomere length. A non-linear relationship was not indicated (*P for non-linearity* = 0.011; Figure 2).

**TABLE 2:** The association between riboflavin intake and telomere length among all/subgroup participants.

Participants	Models	β and 95% CI	*P*-value	*P*^a^ for interaction
All participants	Model 1	0.011 (0.001, 0.021)	0.037	–
	Model 2	0.012 (0.002, 0.023)	0.033	
	Model 3	0.014 (−0.002, 0.029)	0.107	
**Sex subgroup**				
Female	Model 1	0.020 (0.005, 0.035)	0.014	0.044
	Model 2	0.019 (0.004, 0.035)	0.020	
	Model 3	0.029 (0.004, 0.054)	0.046	
Male	Model 1	0.012 (0.000, 0.024)	0.071	
	Model 2	0.008 (−0.005, 0.021)	0.240	
	Model 3	−0.001 (−0.019, 0.017)	0.935	
**BMI subgroup**				
Normal (<25 kg/m^2^)	Model 1	0.005 (−0.010, 0.020)	0.501	0.029
	Model 2	0.001 (−0.016, 0.018)	0.928	
	Model 3	0.007 (−0.024, 0.037)	0.684	
Overweight (25–29.9 kg/m^2^)	Model 1	0.015 (−0.001, 0.030)	0.070	
	Model 2	0.019 (0.002, 0.036)	0.040	
	Model 3	0.005 (−0.018, 0.027)	0.695	
Obese (30–34.9 kg/m^2^)	Model 1	0.013 (−0.008, 0.034)	0.230	
	Model 2	0.012 (−0.014, 0.038)	0.384	
	Model 3	0.003 (−0.050, 0.054)	0.906	
Severe-obese (≥35 kg/m^2^)	Model 1	0.010 (−0.006, 0.026)	0.236	
	Model 2	0.012 (−0.008, 0.031)	0.254	
	Model 3	0.043 (0.014, 0.072)	0.027	

*CI, confidence interval; BMI, body mass index; Model 1 included only the exposure variable, riboflavin intake; Model 2 was additionally adjusted for age, sex, and ethnicity; Model 3 was further adjusted for education levels, smoking, alcohol consumption, BMI, vitamin C intake, vitamin B12 intake, selenium intake, zinc intake, copper intake, dietary fiber intake, coronary heart disease, congestive heart failure and diabetes mellitus.*

*^a^P value for interaction of the Model 3.*

We performed subgroup analyses to assess whether the association between RF intake and telomere length was influenced by sex or obesity level. Participants were divided into two groups based on their sex (male, female) and four groups based on their obesity level (normal, overweight, obese, severe-obese). Significant association between RF intake and telomere length was observed in the female subgroup (Model 1: β = 0.020; 95% CI: 0.005, 0.035; *P* = 0.014; Model 2: β = 0.019; 95% CI: 0.004, 0.035, *P* = 0.020). Even after adjusting for all related factors, the association is still significant (β = 0.029; 95% CI: 0.004, 0.054; *P* = 0.046). In contrast, the association between RF intake and telomere length was not significant in male subgroup for all three models. Furthermore, the RF intake by sex subgroup interaction term was significant (*P_*interaction*_* = 0.044; Table 2).

In terms of obesity level, dietary RF intake was insignificantly associated with telomere length after adjusting for relevant variables in normal, overweight and obese subgroups (β = 0.007; 95% CI: −0.024, 0.037, *P* = 0.684; β = 0.005; 95% CI: −0.024, 0.027, *P* = 0.695; β = 0.003; 95% CI: −0.050, 0.054, *P* = 0.906). However, RF intake and telomere length were significantly positively correlated in the severe obese group (β = 0.043, 95% CI: 0.014, 0.072, *P* = 0.027). The RF intake by BMI subgroup interaction term was significant (*P_*interaction*_* = 0.029; Table 2).

**FIGURE 3:**
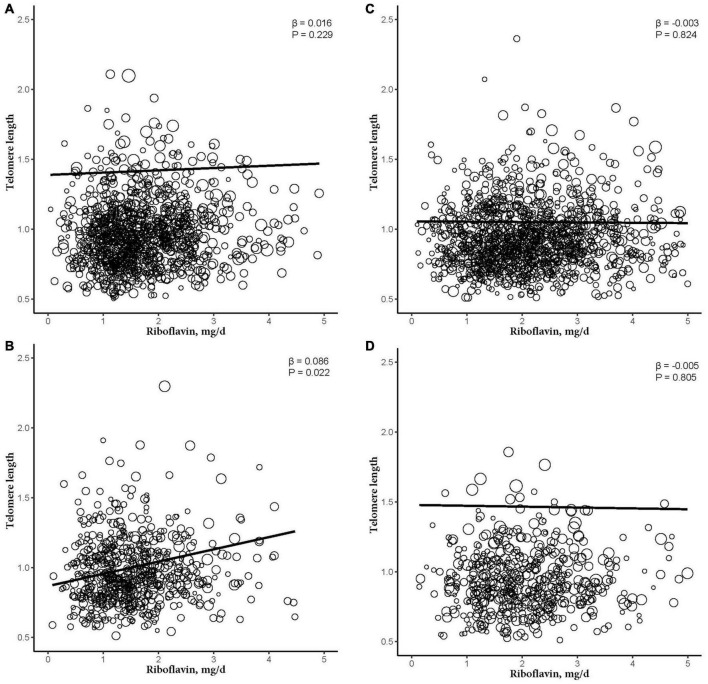
The dose-response relationship between riboflavin intake and telomere among sex and obese subgroup. **(A)** Non-obese female participant. **(B)** Obese female participant. **(C)** Non-obese male participant. **(D)** Obese male participant. Models were adjusted for age, ethnicity, education levels, smoking, alcohol consumption, BMI, vitamin C intake, vitamin B12 intake, selenium intake, zinc intake, copper intake, dietary fiber intake, coronary heart disease, congestive heart failure, and diabetes mellitus.

**FIGURE 4:**
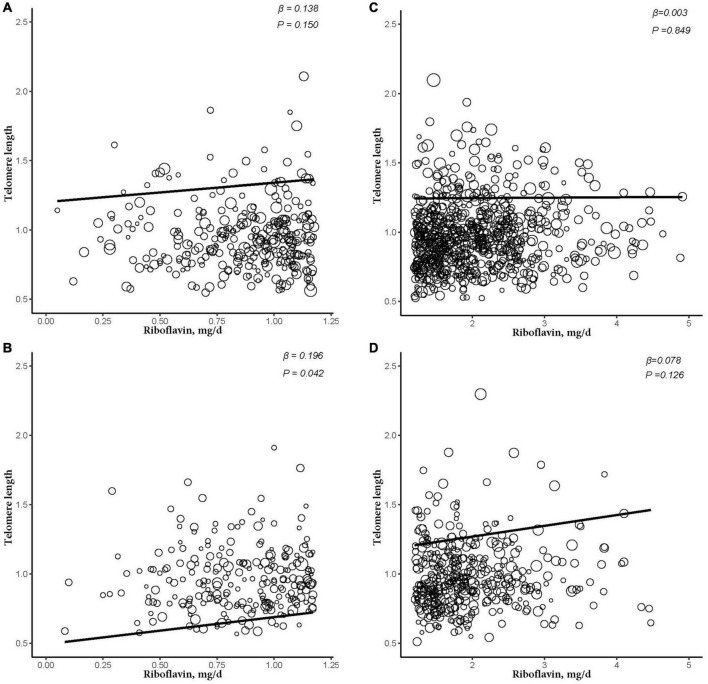
The dose-response relationship between riboflavin intake and telomere in different riboflavin levels among female and obese subgroup. **(A)** Low riboflavin intake and non-obese group. **(B)** Low riboflavin intake and obese group. **(C)** Normal riboflavin intake and non-obese group. **(D)** Normal riboflavin intake and obese group. Models were adjusted for age, ethnicity, Vitamin C intake, smoking, and alcohol consumption.

After further dividing into four groups based on obesity level in the sex subgroups (non-obese: BMI < 30 kg/m^2^; obese group: BMI ≥ 30 kg/m^2^), dietary RF intake was insignificantly associated with telomere length after adjusting for relevant variables in two subgroups of the male subgroup (non-obese: β = −0.003, 95% CI: −0.025, 0.020, *P* = 0.824; obese: β = −0.005, 95% CI: −0.044, 0.034, *P* = 0.805). In the female subgroup, non-obese group showed insignificant association between telomere length and RF intake (β = 0.016, 95% CI: −0.009, 0.041, *P* = 0.229). However, there were significant linear relationships between telomere length and RF intake in the obese group (β = 0.086, 95% CI: 0.027, 0.146, *P* = 0.022; Figure 3). And after further dividing into two groups based on RF intake level in the female subgroups, telomere lengths are significantly associated with RF intake in the female’s obese group (β = 0.196, *P* = 0.042; Figure 4).

## Discussion

In the current investigation, we assessed the association between dietary RF intake and telomere length in a large, nationally representative dataset including 3,788 middle-aged and older Americans. Our findings suggested that there was a significant correlation between dietary RF intake and telomere length in all participants (unadjusted and after adjusted for age, sex, ethnicity). However, after adjusting some other relevant variables, the results became insignificant. Nevertheless, correlations were still significant in female and severe obese participants. And this relationship remained significant after correcting for relevant confounding variables. Meanwhile, there’s a strong positive association between one’s RF intake and telomere length in these participants with inadequate intake.

Telomeres are special terminal regions of chromosomes that do not encode any proteins. However, they protect chromosomes from damage during cell division (24). And it was considered to be a reliable biomarker for predicting aging (25). The telomere length, although highly heterogeneous and genetic (26), has been proposed as a biomarker of aging, survival and health such as cancer, cardiovascular disease (27). Lifestyle, including dietary patterns such as the Mediterranean diet, may affect telomere length (18, 28). Recently, increasing research has shown that nutrient intake was able to influence telomere length. Lin et al. and Shu et al. suggested that higher intake of both copper and selenium was positively associated with telomere length (29, 30). In a study by Pusceddu et al. supplementation with vitamins such as folic acid, vitamin D and vitamin B were also associated with telomere length (31). However, the relationship between dietary RF intake and telomere length has not been elucidated yet. The results of this study suggested that dietary intake of RF is associated with telomere lengthening in middle-aged and older adults in the United States (adjusted for age, sex, and ethnicity). There were several studies that supported our conclusions. Zou et al. had demonstrated that RF supplementation significantly extended the life span of Drosophila and enhanced reproduction by enhancing the activity of antioxidant enzymes (3). In another study in China, dietary RF intake was inversely associated with cancer risk (32). To our knowledge, there were no studies on the relationship between dietary RF intake and telomere length, and our findings may add to the literature on the correlation between dietary factors and telomere length.

The biological mechanisms underlying the association between dietary RF intake and telomere length are not fully understood, and it may be related to the antioxidant effects of RF. RF is essential for nutrient metabolism and antioxidant protection. However, the antioxidant capacity of RF in humans may be far underestimated, as it acts as a coenzyme for oxidoreductase in the FAD and Flavin Mononucleotide (FMN) forms. Several reports have elucidated how RF status affects the activity of antioxidant enzymes. Wang et al. concluded that RF could increase superoxide dismutase activity such as heme oxygenase-1 in heart tissue thus improving the oxidative status of the heart (33). Simultaneously RF status can also affect the activity of other antioxidant enzymes, including glutathione peroxidase (GPx) and catalase. Some animal studies have shown that RF achieves its antioxidant goal by affecting GPx activity. It has been suggested that RF deficiency may cause an increase in lipid peroxidation, resulting in increased GPx activity (34). The possible metastable effect of reactive oxygen species on GPx enzymes could lead to increased activity by increasing lipid peroxidation (35). The effect of RF on lipid peroxidation has also been confirmed in several human studies (36, 37).

Our findings indicated that dietary RF intake is significantly associated with telomere length prolongation in female (adjusted for age, sex, BMI, smoking, alcohol consumption, vitamin C intake, vitamin B12 intake, selenium intake, zinc intake, copper intake, dietary fiber intake, CHF, CHD, and DM), but not in male. This is consistent with the findings of Xu et al., who found that multivitamin use was associated with longer telomere length in females (16). Increased RF intake were also found in several studies to have a significant sex difference in reducing the risk of DM and cardiovascular disease (38, 39), typically higher effect in female than in male. In addition, previous studies have shown a positive correlation between estradiol and antioxidant glutathione peroxidase activity, suggesting that estrogen may help decrease GPx activity in females (40). In another study, Bourgonje et al. concluded that postmenopausal females have relatively high levels of systemic oxidative stress (41). Since the female population in this study is mainly middle-age or older postmenopausal females, RF helped suppress the high oxidative stress levels among them. Moreover, it was interesting to note that the intake of RF was significantly lower in female than in male in our study. The recommended daily intake of RF is 1.10 mg for females (42), our study found that the first quartile daily RF intake among studied females was 1.18 mg, suggesting that around a quarter of the studies female population was prone to insufficient RF intake. Our analysis also showed higher β values for both RF and telomere length among females with obesity and low RF intake (lower than 1.18 mg/day). Therefore, we believe insufficient RF intake may influence telomere length in females with obesity and recommend higher RF intake for females. Nevertheless, our study suggests that females may benefit from additional dietary vitamin B2 supplements.

Previous studies showed that obese subjects have significantly shorter telomere lengths than non-obese subjects (17, 23). Our subgroup analysis on obesity level provides insights for this discussion. The results showed that dietary RF intake was significantly and positively associated with telomere length in severe-obese subjects. However, such association was not observed in non-obese participants. Previous studies showed higher levels of inflammation in obese individuals compared to normal weight individuals, along with similarly elevated levels of oxidative stress (43–45). Furukawa et al. concluded that accumulated fat in obese patients undergoes more oxidative stress (46). Niemann et al. (47) suggested that oxidative stress occurs whenever the release of reactive oxygen species (ROS) exceeds the endogenous antioxidant capacity. Metabolic alterations observed in the hearts of obese patients, such as increased fatty acid oxidation, mitochondrial dysfunction, glucose autoxidation, impaired polyol metabolism, or increased hexosamine metabolism, can lead to increased ROS release (47). RF was an underrated water-soluble vitamin against oxidative stress. RF may reduce inflammation levels or oxidative stress in the obese population. Consequently, RF intake may have a protective effect on telomere length in the obese population. Our results provided support for this hypothesis.

Furthermore, as observed in our study, after further subdividing the sex subgroup into non-obese and obese groups, it was found that telomere length in the female non-obese subgroup was not associated with RF intake, whereas the increase in telomere length in the female obese subgroup was significantly associated with a higher intake of RF. After further analysis, this relationship may be observed in low RF intake participants. The findings of the study by Nordfjäll et al. resonate with our results, by concluding that telomere length seem to be associated with the “obese phenotype,” but only in female (48). Middle-aged and elderly female have decreased estrogen levels, and simultaneously obese people have fatter cell accumulation. Both lead to higher levels of oxidative stress, prompting a more significant telomere length-protective effect of RF. Although, how RF intake affects telomere length in obese individuals and female remains to be further explored. However, our study would still suggest the need for a higher intake of riboflavin-rich diets or even direct RF supplementation among the middle-aged and older female low RF intake population, especially those with obesity.

There are several strengths of this study that are worth noting. First, telomere length was measured in the laboratory using well-established methods, which ensured the accuracy of our measurements. Second, this study was based on a widely renown and acknowledged survey (NHANES) that sampled the population in a strictly random process, and therefore, these results are representative of the whole population. Finally, the statistical regression model took into account several potential confounders, including age, sex and ethnicity. However, several limitations should also be noted. First, as a cross-sectional study, the design of NHANES provides no indication for causal or temporal associations. Second, the nature of the 24-h dietary recall interviews causes inevitable inaccuracies for dietary intake measurements brought by mis-memorization, but we tried to minimize this bias by excluding those who cannot be interviewed within 24 h and those under the minimum criteria on dietary recall status. Third, we cannot exclude the influence of anti-oxidating potentials on the results. Forth, we did not investigate the intake of nutrient supplements, which may bias the results of the study. Finally, we did not explore the relationship between serum RF levels and telomere length. Similar studies in the future could contribute to further understanding of the biological mechanism through explaining the relationship between blood RF levels and telomere length.

## Conclusion

This study showed that increased dietary RF intake was positively associated with longer telomere length in middle-aged and older American female and obese populations. Although our findings are based on a cross-sectional study, our research may remind females with obesity and insufficient RF intake of the potential benefits of RF intake on preserving telomere length. In addition, this may enhance our understanding on disease diagnosis and post-treatment recovery. Our analysis also sheds lights on the mechanisms of diseases related to aging organisms and other metabolic disorder.

## Data Availability Statement

The datasets presented in this study can be found in online repositories. The names of the repository/repositories and accession number(s) can be found below: https://wwwn.cdc.gov/nchs/nhanes/Default.aspx.

## Ethics Statement

The studies involving human participants were reviewed and approved by the Ethics Review Board of the NCHS (Protocol #98e12). The patients/participants provided their written informed consent to participate in this study.

## Author Contributions

WC participated in formulating the research question, design of analyses, interpretation of the data, drafting and revising the manuscript, and approval of the final version. SS participated in the design of analyses, data analysis, revising the manuscript, and approval of the final version. YJ interpretated the data, drafted and revised the manuscript, and approved the final version of the manuscript. LC revised the manuscript and approval of the final version. YL participated in formulating the research question, design of analyses, interpretation of the data, revising the manuscript, and approval of the final version. KC participated in formulating the research question, design of analyses, revising the manuscript, and approval of the final version. KH participated in formulating the research question, design of analyses, data analysis, interpretation of the data, and approval of the final version. All authors read and approved the final version of the manuscript and are responsible for all aspects of the manuscript.

## Conflict of Interest

The authors declare that the research was conducted in the absence of any commercial or financial relationships that could be construed as a potential conflict of interest. The reviewer YL declared a shared affiliation with the author YJ to the handling editor at the time of review.

## Publisher’s Note

All claims expressed in this article are solely those of the authors and do not necessarily represent those of their affiliated organizations, or those of the publisher, the editors and the reviewers. Any product that may be evaluated in this article, or claim that may be made by its manufacturer, is not guaranteed or endorsed by the publisher.

## Footnotes

1
cdc.gov

2
https://wwwn.cdc.gov/nchs/nhanes/continuousnhanes/default.aspx?BeginYear=1999
