# Supplementary material for: Association Between Riboflavin Intake and Telomere Length: A Cross-Sectional Study From National Health and Nutrition Examination Survey 1999–2002
Source: Front Nutr. 2022 Mar 31;9:744397. doi: 10.3389/fnut.2022.744397 (PMC9009291; doi:10.3389/fnut.2022.744397)
Supplement: Supplementary file 1 [file Table_1.pdf]

Supplementary table 1. The correlation coefficients between riboflavin and other anti-oxidants.

	Riboflavin
Vitamin C	0.29*
Copper	0.35*
Zinc	0.38*
Selenium	0.43*
Dietary fiber	0.43*
Vitamin B12	0.44*
Vitamin E	0.50*
Magnesium	0.60*
Niacin	0.64*
Potassium	0.64*
Total Folate	0.69*
Iron	0.70*
Vitamin B6	0.71*
Thiamine	0.74*
Calcium	0.77*
